# Switching iron sucrose to ferric carboxymaltose associates to better control of iron status in hemodialysis patients

**DOI:** 10.1186/s12882-018-1045-8

**Published:** 2018-09-20

**Authors:** Jesse M. G. Hofman, Michele F. Eisenga, Adry Diepenbroek, Ilja M. Nolte, Bastiaan van Dam, Ralf Westerhuis, Stephan J. L. Bakker, Casper F. M. Franssen, Carlo A. J. M. Gaillard

**Affiliations:** 1 Department of Nephrology, University Medical Center Groningen, University of Groningen, Hanzeplein 1, Groningen, 9713GZ The Netherlands; 2 Department of Epidemiology, University Medical Center Groningen, University of Groningen, Hanzeplein 1, Groningen, 9713GZ The Netherlands; 3 Department of Internal Medicine, Medical Center Alkmaar, Wilhelminalaan 12, 1815JD Alkmaar, The Netherlands; 4 Dialysis Center Groningen, Hanzeplein 1, Groningen, 9713GZ The Netherlands; 5 Department of Internal Medicine and Dermatology, University Medical Center Utrecht, University of Utrecht, Heidelberglaan 100, 3584CX Utrecht, The Netherlands; 6 Department of Internal Medicine, Division of Nephrology, University Medical Center Groningen, Hanzeplein 1, 9713GZ Groningen, The Netherlands

**Keywords:** Ferric carboxymaltose, Iron sucrose, Hemodialysis, Iron status, ESA

## Abstract

**Background:**

Although the efficacy of iron sucrose (IS) and ferric carboxymaltose (FCM) in treating anemia in hemodialysis (HD) patients has been studied individually, a comparison of these two intravenous iron formulations has not yet been performed in HD patients.

**Methods:**

We performed a retrospective audit on records of 221 stable HD patients from different HD centers in the Netherlands, who were switched from IS to FCM on a 1:1 ratio. To assess the effect of the switch on iron status parameters, data from 3 time points before and 3 time points after the switch were analyzed using linear mixed effects models. Subanalyses were done in 2 subgroups of patients anemic or iron deficient at baseline.

**Results:**

Hemoglobin increased in all groups (anemic [1.4 g/dL, *P* < 0.001] iron deficient [0.6 g/dL, *P* < 0.001]), while the weekly iron dose was significantly lower when patients received FCM compared to IS (48 vs 55 mg/week, *P* = 0.04). Furthermore, serum ferritin and transferrin saturation increased in all groups (anemic [64 μg/L, 5.0%, *P* < 0.001] iron deficient [76 μg/L, 3.6%, *P* < 0.001]). Finally, the darbepoetin α dose decreased significantly in all groups (anemic [− 16 μg/wk., *P* = 0.01] iron deficient [− 11 μg/wk., *P* < 0.001]).

**Conclusions:**

In this real-life study in HD patients, a switch from IS to FCM resulted in an improvement of iron status parameters despite a lower weekly dose of FCM. Furthermore, the ESA dose was reduced during FCM, while hemoglobin levels increased.

**Electronic supplementary material:**

The online version of this article (10.1186/s12882-018-1045-8) contains supplementary material, which is available to authorized users.

## Background

Anemia is a frequent complication of chronic kidney disease (CKD) [1]. In CKD, the main causes of anemia are deficiency of erythropoietin, iron-restricted erythropoiesis and anemia of the chronic disease (ACD) [2–4]. The latter originates from the chronic inflammation that is a hallmark of CKD patients and has been shown to be associated with adverse outcomes such as cardiovascular events, end-stage renal disease, increased mortality, and decreased quality of life [5]. In ACD, pro-inflammatory cytokines upregulate hepcidin production in the liver which subsequently hampers iron uptake from the gut and iron release from the reticulo-endothelial system [6, 7] which leads to functional iron deficiency that negatively affects erythropoiesis. Furthermore, increased iron utilization due to the use of erythropoiesis stimulating agents (ESA), and iron loss as a result of dialysis-related blood loss contribute to the high prevalence of anemia in patients with CKD [8].

Oral administration of iron has limited efficaciousness and is associated with gastrointestinal side effects. By means of intravenous iron, gastrointestinal absorption is bypassed and incorporated more rapidly [5]. Indeed, it has been established that intravenous iron supplementation as a treatment for iron deficiency anemia is superior to oral iron supplementation in non-dialysis dependent CKD, hemodialysis (HD) and peritoneal dialysis patients [9–11]. Furthermore, ESA requirements have been shown to be decreased in patients receiving intravenous iron [12].

Nowadays, several intravenous iron supplementations are available, of which iron sucrose (IS) and a more recently introduced intravenous (IV) iron compound, ferric carboxymaltose (FCM) (brand names Venofer and Ferinject, respectively) are frequently used. Although the efficacy of IS and that of FCM for treatment of anemia in CKD patients have been studied individually by comparing the formulations to oral iron supplementation [10, 13], a head to head comparison of these two intravenous formulations has never been performed in HD patients. Therefore, the goal of this audit is to analyze the effects of a switch from IS to equally dosed FCM. Hence, we performed an audit on records of HD patients in three dialysis centers that switched from IS to FCM.

## Methods

### Study design

**Fig. 1:**
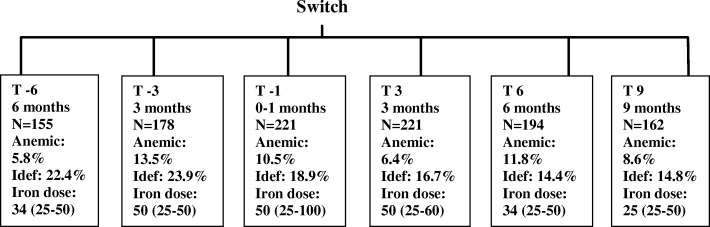
Graphical representation of the time points used for data collection from patient records. The figure shows the amount of patients included at each timepoint, the percentage of people included in the anemic (Hemoglobin < 10 g/dL) and iron deficient (TSAT < 20% and Ferritin < 300 μg/L) subgroups, and the median (IQR) iron dose (mg/week) at each timepoint. Idef, iron deficient

We conducted an audit using data retrospectively gathered from HD patients who were switched from IS to FCM because of a change in hospital policies. Patients from four dialysis centers were included (Dialysis Center Groningen, *n* = 110, University Medical Center Groningen, *n* = 11, Dialysis Center “Noord-West Ziekenhuizgroep”, *n* = 54, and Dialysis Center Amersfoort, *n* = 46). We analyzed a study period of 15 months in total, during which we studied 6 time points, each 3 months apart, as illustrated in Fig. 1. During the 6 months pre-switch patients received IS, and during the 9 months post switch they received FCM. During both periods, blood was sampled every 3 months as part of the clinical routine, at least one week after administration of IV iron. The baseline time point was one of the three time points before the switch, and was defined for each patient separately when the patient had at that time point been using maintenance IV iron medication for ≥6 months, and the patient had been on HD treatment for at least 3 months. The need for participant consent was waived by the medical ethical committee in the UMCG for the act on Medical Research Involving Human Subjects (in Dutch:WMO).

### Study population

The study population consisted of CKD patients who were at least 18 years old, were on uninterrupted HD treatment for a period of at least 3 months before the switch and 3 months after the switch, were using IS at the start of the investigational period, and were subsequently switched from IS to FCM. Patients with any malignancy diagnosed in the five years prior to the investigational period were excluded. In all three cohorts anemia was diagnosed when hemoglobin was < 12 g/dL. Subsequently a diagnostic workup was performed to rule out causes of anemia besides CKD, like bleeding or active infection. If these were ruled out, IV iron therapy was initiated when there was an absolute iron deficiency (transferrin saturation (TSAT) < 20% and ferritin < 100 ng/mL) or when TSAT was ≤20%, or ferritin was ≤200 μg/l, and an increase in hemoglobin concentration was desired without increasing the ESA dose. In this case, the patient received 100 mg of iron per week for 10 weeks. Subsequently, iron medication was ceased, and after 4 weeks the patient’s iron stores were assessed by the treating nephrologist, who decided on the treatment plan. If ferritin was < 200 μg/l, or TSAT was < 20%, IV iron was initiated at a dose of 100 mg per week. If ferritin was 200–500 μg/l, and/or TSAT was 20–30%, the dose was 100 mg every two weeks. If ferritin was 500–800 μg/l and/or TSAT was 30–50%, the dose was 100 mg every four weeks. If ferritin was > 800 μg/l and/or TSAT was > 50%, no IV iron was administered. If IV iron alone was not sufficient to improve hemoglobin levels, the treating nephrologist added an ESA to the therapeutic regime. In all cases, the patient was re-evaluated every three months, at least a week after the last administration of IV iron. The goal of this treatment was to prevent hemoglobin from dropping below 10 g/dL, while not routinely exceeding 12 g/dL. Iron was always administered in the final 30 min of the HD session. The protocol did not change after switching from IS to FCM. This is in accordance with the 2015 Dutch National federation for Nephrology guidelines for management of anemia in kidney disease that are based on the most recent KDIGO guidelines and the ERBP position statement on the KDIGO guidelines [14].

After application of the exclusion criteria, data from 221 of the original 280 patients who were switched from IS to FCM were included in the final analyses. Following inclusion of data from the baseline time point, data was included for analysis up until the final time point 9 months after the switch, or until follow-up was censored because the patient died, stopped using FCM, or stopped HD treatment due to any cause. As depicted in Fig. 1, at T-6 data from 155 patients were included, at T-3 data from 178 patients were included, and at T-1, by definition, data from all 221 patients were included. After inclusion, all patients were followed up until T3, the first time point, i.e. 3 months after the switch, 194 patients were followed up until T6, i.e. 6 months after the switch, and 162 until T9, i.e. 9 months after the switch. As a result, we analyzed the data with linear mixed models, which is considered to be robust to missing values and is therefore able to deal with missing data at certain time points. We also assessed the effect of the factor “time” on the period from T-6 to T-1 (patients on IS) and T3 to T9 (patients on FCM).

### Audit

The primary objective of this analysis was to determine whether the switch from IS to FCM modulated iron status parameters in relation to iron dose and ESA dose. We used the parameters TSAT and serum ferritin over time to quantify the effect on both absolute and functional iron deficiency, as they have been shown to be key parameters in the diagnosis of iron deficiency anemia (IDA) [15], and hemoglobin over time to determine the effect on the severity of anemia. To further address other effects of the switch on erythropoiesis, iron metabolism and inflammation, we included ESA dose, iron dose, serum iron, mean corpuscular volume (MCV), hematocrit, reticulocyte count, total iron-binding capacity and C-reactive protein (CRP) as secondary objectives. All data was gathered from blood samples that were analyzed at the main laboratory in each hospital.

### Statistical analysis

Normally distributed variables are described as mean ± standard deviation (SD), and skewed variables as median and interquartile range (IQR). Categorical variables are expressed as percentage. Linear mixed effects models, which are within-person analyses, were used to assess the effect of the medication switch on iron status and dialysis parameters. Random effects were patient identification number (ID) and an interaction between ID and switch. The most appropriate covariance structure was determined for each outcome variable separately. Fixed effects were switch, factor time classified as three options for the three time moments before and after switch, time^2^ to account for the possible nonlinear relation between time and the outcome variable, iron deficiency at baseline, anemia at baseline, and interactions between switch and anemia, switch and iron deficiency, switch and time, and switch and time^2^. The least significant fixed effects were removed in a stepwise manner for each variable, until only effects with a significance of < 0.1 were left in order to achieve a good model fit. Subsequently, the estimated marginal means of parameters before and after the switch were compared to determine the main effect of the switch. Skewed variables were ln-transformed in order to achieve a normal distribution. Subanalyses were performed in a subgroup of iron deficient patients, characterized by TSAT < 20% and ferritin < 300 μg/L (*n* = 55), and anemic patients, characterized by a hemoglobin of < 10 g/dL in men and women [16], which was the lower end of the hemoglobin target range of 10–12 g/dL (*n* = 24). Data analysis was performed in SPSS statistics version 22.0 (IBM Corporation, Chicago, IL, USA).

## Results

We included 221 HD patients (mean age 65 ± 15 years), of whom 24 were anemic at baseline, and 55 iron deficient at baseline. The prevalence of hypertension and diabetes mellitus was 58.6% and 31.8%, respectively. There was no significant correlation between ferritin and CRP at baseline in the entire group (β − 0.090, *P* = 0.33), the anemic group (β 0.286, *P* = 0.32), or the iron deficient group (β − 0.128, *P* = 0.64). Additional baseline characteristics of the entire group, as well as anemic and iron deficient subgroups are shown in Table 1.

**Table 1:** Baseline characteristics of patients included in the study

	All patients *n* = 221	Anemic *n* = 24	Iron deficient *n* = 55
Sex (% male)	62.3	45.5	67.3
Age (years)	65 ± 15	62 ± 16	64 ± 18
Diabetes mellitus (%)	31.8	35.0	20.8
Hypertension (%)	58.6	65.0	54.2
BMI (kg/m^2^)	24.7 ± 4.1	25.4 ± 4.5	25.0 ± 3.7
Systolic BP pre-HD (mmHg)	140 ± 24	135 ± 26	141 ± 21
Diastolic BP pre-HD (mmHg)	69 ± 14	70 ± 15	69 ± 13
Residual diuresis (%)	47.6	37.5	42.9
Ultrafiltration (L)	2.15 ± 0.96	2.55 ± 0.76	1.98 ± 0.92
Dialysis vintage (months)	22 (11–49)	22 (10–44)	26 (9–55)
Urea pre-HD (mg/dL)	23.2 ± 7.2	21.8 ± 7.8	24.5 ± 6.9
Creatinine pre-HD (μg/dL)	808 ± 250	842 ± 257	870 ± 259
Hb (g/dL)	11.7 ± 1.2	9.4 ± 0.5	11.8 ± 1.3
Ht	0.35 ± 0.03	0.30 ± 0.02	0.36 ± 0.04
Reticulocytes (%)	13.7 (10.3–19.0)	14.0 (12.0–22.3)	14.0 (12.0–18.0)
MCV (fL)	95.4 ± 6.4	96.8 ± 6.9	91.0 ± 5.6
Ferritin (μg/L)	416 (227–625)	514 (297–700)	176 (116–226)
Transferrin (g/L)	1.9 ± 0.3	1.9 ± 0.4	2.1 ± 0.3
TSAT (%)	20.8 (15.0–26.5)	18.5 (14.0–31.3)	14.0 (12.0–16.0)
Serum iron (μg/L)	9.3 (7.0–12.8)	9.0 (7.0–14.0)	7.0 (6.0–8.0)
TIBC (μmol/L)	46.0 ± 9.2	44.3 ± 9.0	49.4 ± 9.6
Iron medication (mg/wk)	50 (25–100)	100 (50–100)	50 (33–100)
Darbepoetin α (μg/wk)	30 (10–50)	40 (50–60)	30 (16–60)
Epoetin β (IE/wk)	8000 (4000–12,000)	8000 (7000–13,500)	8000 (4000–12,000)
CRP (mg/L)^a^	6.2 (2.0–16.0)	13.5 (2.0–40.0)	15.0 (1.5–56.0)
Phosphate (mmol/L)	1.59 (1.29–1.93)	1.73 (1.31–2.16)	1.61 (1.3–2.0)
Potassium (mmol/L)	4.9 (4.5–5.4)	4.8 (4.5–5.6)	5.0 (4.6–5.5)
Sodium (mEq/L)	138.0 ± 3.1	138.7 ± 2.8	137.3 ± 3.3

Normally distributed variables are described as mean ± SD and variables with a skewed distribution as median, IQR. Dialysis duration and frequency are described as mode

*BP*, blood pressure; *HD*, hemodialysis; *Hb*, hemoglobin; *Ht*, hematocrit; *MCV*, mean corpuscular volume; *TSAT*, transferrin saturation; *TIBC*, total iron binding capacity; *CRP*, C-reactive protein

^a^Data available in 119 HD patients before the switch

### Effects of the switch in the entire cohort

**Fig. 2:**
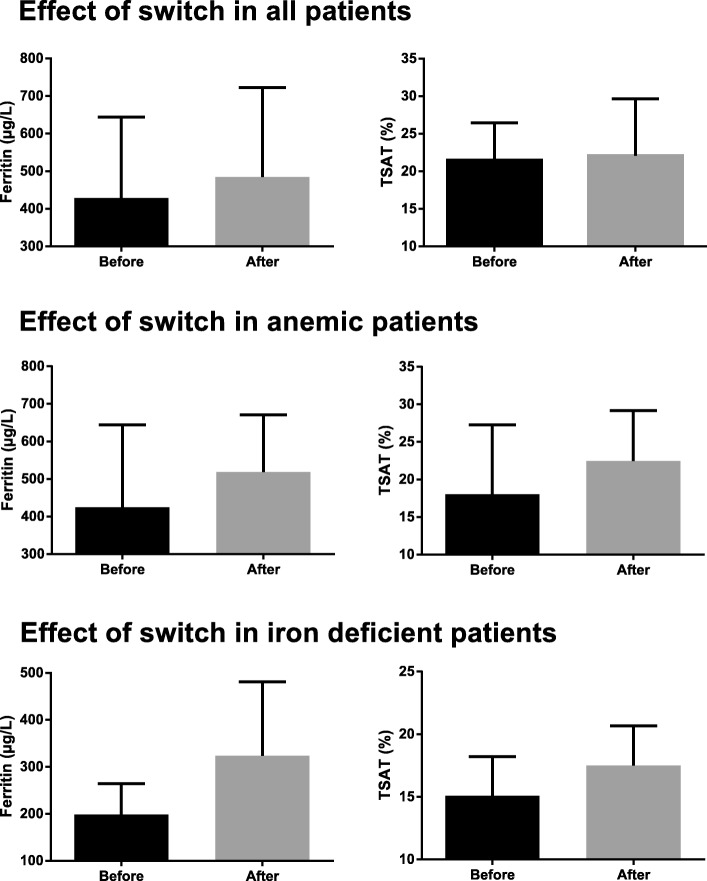
Bar charts showing the median serum ferritin and TSAT levels of the three time points before and after the switch for the entire population and the 2 subgroups. Data is described as median and interquartile range

The models used to analyze the effect of the switch on each parameter are described in Additional file 1: Table S1. The main effects of the switch from IS to FCM on all parameters are reported in Table 2. The dosage of iron medication decreased significantly after switch from IS to FCM (− 7 mg/wk., *P* = 0.04), while hemoglobin (0.64 g/dL, *P* < 0.001) and hematocrit (0.02, *P* < 0.001) increased significantly. Furthermore, after the switch from IS to FCM, serum ferritin increased significantly (64 μg/L, *P* < 0.001) as well as TSAT (3.4%, *P* < 0.001) (Fig. 2). The proportion of reticulocytes in the blood and serum transferrin decreased significantly (− 0.1 g/L, *P* = 0.002). The factor time was not a significant predictor of TSAT and serum ferritin both before and after the switch; hence the data shown below are fully the result of the switch. The ESA dose was decreased significantly in patients using darbepoetin α, but not in those using epoetin β. MCV showed a slight significant increase. Other parameters did not change significantly as result of the switch of IS to FCM.

**Table 2:** Main effect of medication switch in the total population and subgroups

	Total population (*n* = 221)			Anemic at baseline (*n* = 24)			Iron deficient at baseline (*n* = 55)		
	Before (SE)	After (SE)	*P*	Before (SE)	After (SE)	*P*	Before (SE)	After (SE)	*P*
Ln Irondose (mg/wk)	4.007 (0.063)	3.880 (0.063)	0.04	4.114 (0.046)	3.987 (0.045)	0.006	4.094 (0.086)	3.967 (0.086)	0.11
Irondose (mg/wk)	55	48		61	54		60	53	
Ln Darbepoetin α (**μ**g/wk)	3.528 (0.122)	3.299 (0.121)	0.001	3.809 (0.230)	3.375 (0.227)	0.01	3.649 (0.155)	3.290 (0.155)	< 0.001
Darbepoetin α (**μ**g/wk)	34	27		45	29		38	27	
Ln Epoetin β (IE/wk)	9.038 (0.099)	8.911 (0.096)	0.06	N/A^a^	N/A^a^	N/A^a^	N/A^a^	N/A^a^	N/A^a^
Epoetin β (IE/wk)	8400	7400		N/A^a^	N/A^a^		N/A^a^	N/A^a^	
Hb (g/dL)	11.00 (0.11)	11.64 (0.10)	< 0.001	10.10(0.196)	11.50 (0.184)	< 0.001	11.21 (0.134)	11.85 (0.130)	< 0.001
Ht	0.338 (0.003)	0.358 (0.003)	< 0.001	0.316 (0.006)	0.358 (0.006)	< 0.001	0.345 (0.004)	0.364 (0.004)	< 0.001
MCV (fL)	94.10 (0.453)	94.92 (0.452)	0.001	94.10 (0.453)	94.92 (0.452)	0.001	91.46 (0.759)	92.28 (0.758)	0.08
Ln Reticulocytes (%)	2.613 (0.036)	2.704 (0.033)	< 0.001	2.613 (0.036)	2.704 (0.033)	0.002	2.613 (0.036)	2.704 (0.033)	0.004
Reticulocytes (%)	13.6	14.9		13.6	14.9		13.6	14.9	
Ln Ferritin (**μ**g/L)	5.659 (0.041)	5.860 (0.055)	< 0.001	5.659 (0.041)	5.860 (0.055)	< 0.001	5.132 (0.071)	5.503 (0.095)	< 0.001
Ferritin (**μ**g/L)	287	351		287	351		169	245	
Ln Transferrin (g/L)	0.640 (0.013)	0.611 (0.014)	0.002	0.640 (0.013)	0.611 (0.014)	0.04	0.702 (0.022)	0.674 (0.023)	0.03
Transferrin (g/L)	1.9	1.8		1.9	1.8		2.0	2.0	
Ln TSAT (%)	2.841 (0.037)	3.019 (0.044)	< 0.001	2.760 (0.067)	3.035 (0.080)	< 0.001	2.609 (0.050)	2.844 (0.062)	< 0.001
TSAT (%)	17.1	20.5		15.8	20.8		13.6	17.2	
TIBC (**μ**mol/L)	47.648 (0.707)	47.225 (0.764)	0.35	47.648 (0.707)	47.225 (0.764)	0.12	50.240 (1.112)	49.818 (1.148)	0.53
Ln Serumiron (**μ**mol/L)	2.13 (0.04)	2.26 (0.04)	0.003	2.08 (0.07)	2.29 (0.08)	0.008	1.96 (0.05)	2.14 (0.06)	0.001
Serumiron (**μ**mol/L)	8.4	9.6		8.0	9.9		7.1	8.5	
Ln CRP (mg/L)	1.999	1.825 (0.092)	0.23	1.999 (0.121)	1.825 (0.092)	0.004	1.999 (0.121)	1.825 (0.092)	0.20
CRP	7.4	6.2		7.4	6.2		7.4	6.2	
Ultrafiltration (L)	2.307 (0.103)	2.214 (0.100)	0.23	2.459 (0.193)	2.235 (0.186)	0.22	2.307 (0.103)	2.214 (0.100)	0.32
Ln Phosphate	0.440 (0.020)	0.416 (0.020)	0.19	0.440 (0.020)	0.416 (0.020)	0.14	0.440 (0.020)	0.416 (0.020)	0.17
Phosphate	1.55	1.52		1.55	1.52		1.55	1.52	

Shown in this table are the marginal estimated means of all data before and after the switch, as calculated with our model. Data is described as mean (SE). *P*-values of paired samples t-tests are shown. Ln-transformed variables were transformed back in order to give insight into the effect size

*SE*, standard error; *Hb,* hemoglobin; *Ht*, hematocrit; *MCV*, mean corpuscular volume; *TSAT,* transferrin saturation; *TIBC*, total iron binding capacity; *CRP*, C-reactive protein

^a^Sample size was too small to draw useful conclusions

### Effects of the switch in anemic patients alone

When assessing the same parameters in specifically anemic patients at baseline, hemoglobin (1.4, *P* < 0.001), hematocrit (0.04 *P* < 0.001), TSAT (5.0%, *P* < 0.001), and serum iron (1.9 μmol/L, *P* = 0.008) seemed to increase more as a result of the switch in this subgroup compared to the entire population, while the darbepoetin α dose decreased more (− 16 μg/wk., *P* = 0.010), as shown in Table 2. The models show that having a hemoglobin level of < 10 g/dL at baseline enhances the effect of the switch beyond the effect the switch has in non-anemic individuals on hemoglobin, hematocrit, TSAT, serum iron, and ESA dose. For the other variables, including ferritin (Fig. 2), results were similar to those described for the entire population.

### Effects of the switch in iron deficient patients alone

In this subgroup, the ESA dose decreased more than in the entire population (darbepoetin α: − 11 μg/wk., *P* < 0.001) (Table 2). Furthermore, it shows that being iron deficient at baseline enhances the effect of the switch on serum ferritin (76 μg/l, *P* < 0.001). For all the other variables the results were similar to those described for the entire population.

## Discussion

In this analysis, we have shown that the switch from IS to FCM in HD patients was associated with a significant improvement of iron status, unrelated to iron dose. After the switch to FCM hemoglobin, hematocrit, serum ferritin, TSAT, and MCV increased, while transferrin levels decreased, reflecting erythropoiesis which is less restricted by iron deficiency [17]. Furthermore, ESA dose decreased after the switch to FCM. This is, to our knowledge, the first investigation to compare the effects on iron status of a switch to FCM from IS in dialysis-dependent CKD patients.

In the subgroup of anemic patients at baseline especially, the effect of the switch on iron status was even more pronounced, despite the fact that these patients were prescribed significantly less iron and a smaller ESA dose after the switch. Switching from IS to FCM resulted in a marked increase in TSAT in both groups, a large increase of MCV in the anemic group, and an increase in serum ferritin in both groups. Although the baseline hemoglobin and iron status in the anemic and iron deficient groups were worse, the large improvement of iron status in these groups seems attributable to the effect of switching from IS to FCM since we did not identify a statistically significant general trend based solitarily on the factor time.

These results seem to indicate that switching from IS to similarly dosed FCM causes an increase in ferritin and TSAT in all groups, which is more pronounced in anemic patients. When speculating on possible mechanisms why FCM seems to be more effective than IS in replenishing iron stores, one could put forth the argument that FCM has increased bioavailability of elemental iron compared to IS. FCM and IS are both composed of an iron(III)-hydroxide core, surrounded by a carbohydrate shell (carboxymaltose and sucrose, respectively) [18]. FCM has a higher molecular weight than IS (150,000 v 43,300 Da) and a longer half-life (7–12 v 5–6 h) which increase the area under the curve, indicating that the bioavailability of FCM is greater than that of IS [18]. A second explanation might be that FCM is much more stable than IS, which prevents release of labile iron into the blood, where it can saturate transferrin and lead to significant amounts of non-transferrin bound iron (NTBI) [19]. This causes not only a less efficient uptake of iron by reticuloendothelial macrophages [18], but the NTBI may also lead to oxidative stress [20]. At this point it should be noted that there is no long-term data available on the pharmacokinetics of both compounds; therefore a direct comparison between the two compounds cannot be performed. A head to head pharmacokinetic study would be required before conclusions can be drawn.

These results are in accordance with the results of the REPAIR-IDA trial, a randomized controlled trial comparing FCM to IS in a group of 2584 non-renal IDA patients. Onken et al. describe a significant increase in serum ferritin and TSAT in favor of FCM, 56 days after drug administration [21]. We have demonstrated that over a period of 9 months, a significant difference in serum ferritin and TSAT continues to exist due to administration of FCM.

One of the adverse effects of FCM described is hypophosphatemia. We observed a nonsignificant 0.03 mmol/L decrease in serum phosphate levels; hence, hypophosphatemia did not become more prevalent as a result of the switch from IS to FCM. This is in accordance with a clinical trial in inflammatory bowel disease patients, which described a transient decrease of serum phosphate levels in the FCM group which resolved between week 4 and week 12 after the switch [22]. Our results contradict the findings of a study by Hardy et al. specifically assessing the effect of FCM on serum phosphate levels compared to IS in iron deficiency anemia patients. The authors found that FCM caused significantly more hypophosphatemia than IS, which resolved after a mean duration of 6 months [23]. It should be noted, however, that the latter study did not comprise solitarily chronic kidney disease patients. Hence, the diminished renal function and as such the lower risk of developing hypophosphatemia in our study might have resulted in a substantive difference between our identified prevalence and the results as described by Hardy and colleagues.

It is known, at least theoretically, that administration of intravenous iron in CKD patients on the long term might lead to an iron overload, which may produce endothelial dysfunction, cardiovascular disease, and immune dysfunction [24]. It seems plausible, based on our results, that we can correct iron status parameters more efficiently with FCM, at a lower dose, and as such a putative iron overload can be prevented. Furthermore, we can also speculate that FCM administration could be more cost-efficient than IS due to the lower iron and ESA dose, which may lead to financial savings in the long term [25]. A well performed cost-benefit analysis is needed to substantiate this possible advantage of using FCM as compared to IS.

A strength of our investigation is that it comprises a comparison of multiple data points within one patient, meaning that our results correspond better to a real-life situation where a patient is switched from IS to FCM. Moreover, it should be kept in mind that we censored patients who stopped using FCM as data was no longer available, likely contributing to the underestimation of the effect of FCM as these patients will likely have iron status parameters longer in target.

A limitation of our study is the longitudinal study design without randomization, making it difficult to draw firm conclusions. A head-to-head comparison between IS and FCM is needed to confirm our results. Furthermore, we acknowledge as limitation that we did not assess the tolerability or safety of either IV iron preparation even though these factors play an important role when prescribing. Although the safety of FCM has never been compared to IS in the HD population specifically, it has been assessed in other patient groups such as non-dialysis dependent CKD in the REPAIR-IDA trial [21]. In this trial, Onken et al. found no significant difference in the number of patients that reached a primary composite safety endpoint. Hypertensive events immediately following drug administration occurred significantly more in the FCM group than in the IS group, with 7.45% of patients compared to 4.36% experiencing an event, however, hypertensive events occurred on non-dosing days nearly twice as often in the IS group.

## Conclusions

In conclusion, the switch from IS to FCM was accompanied by a marked improvement in iron status parameters, despite a lower iron dose. In addition, use of FCM resulted in an increase in hemoglobin levels while ESA dose was decreased. Our results need to be confirmed and delineated in more detail in larger prospective studies.

## Additional file

Additional file 1: **Table S1.** Effects of switch, anemia at baseline, and iron deficiency at baseline in individual patients, as analyzed with mixed models analyses. This table shows the model used for the analysis of the effects of the medication switch, iron deficiency at baseline, and anemia at baseline on all parameters. (DOCX 21 kb)

## Abbreviations

ACD: Anemia of chronic disease
CKD: Chronic kidney disease
CRP: C-reactive protein
ESA: Erytrhropoiesis-stimulating agents
FCM: Ferric carboxymaltose
HD: Hemodialysis
ID: Identification number
IDA: Iron deficiency anemia
IQR: Interquartile range
IS: Iron sucrose
IV: Intravenous
MCV: Mean corpuscular volume
NTBI: Non-transferrin bound iron
SD: Standard deviation
TIBC: Total iron binding capacity
TSAT: Transferrin saturation
