# Supplementary material for: Switching iron sucrose to ferric carboxymaltose associates to better control of iron status in hemodialysis patients
Source: BMC Nephrol. 2018 Sep 20;19:242. doi: 10.1186/s12882-018-1045-8 (PMC6149056; doi:10.1186/s12882-018-1045-8)
Supplement: Supplementary file 1 — Table S1. Effects of switch, anemia at baseline, and iron deficiency at baseline in individual patients, as analyzed with mixed models analyses. This table shows the model used for the analysis of the effects of the medication switch, iron deficiency at baseline, and anemia at baseline on all parameters. (DOCX 21 kb) [file 12882_2018_1045_MOESM1_ESM.docx]

**Table S1.** Effects of switch, anemia at baseline, and iron deficiency at baseline in individual patients, as analyzed with mixed models analyses.

|  | Basic effect of switch in patient without anemia or iron deficiency | | | Additional effect of anemia at baseline on switch (Switch*A) | | | Additional effect of iron deficiency at baseline on switch (Switch*Idef) | | | Covariance Structure |
| --- | --- | --- | --- | --- | --- | --- | --- | --- | --- | --- |
|  | Effect | SE | P | Effect | SE | P | Effect | SE | P |  |
| Hb (g/dL) | -.12 | .08 | .12 | 1.51 | .24 | **<.001** | / | / | / | CS |
| Ht | -.003 | .003 | .27 | .045 | .008 | **<.001** | / | / | / | CS |
| MCV (fl) | .82 | .25 | **.001** | / | / | / | / | / | / | CS |
| Ln Reticulocytes (%) | .36 | .15 | **.02** | / | / | / | / | / | / | CS |
| Ln Ferritin (µg/L) | .030 | .047 | .53 | / | / | / | .341 | .093 | **<.001** | TP |
| Ln Transferrin (g/L) | -.029 | .009 | **.001** | / | / | / | / | / | / | TP |
| Ln TSAT (%) | .022 | .076 | .52 | .194 | .087 | **.03** | .116 | .062 | .06 | TP |
| TIBC (µmol/L) | 3.95 | .91 | **<.001** | / | / | / | / | / | / | TP |
| Ln Serumiron (µmol/L) | -.01 | .03 | .67 | .18 | .08 | **.04** | .11 | .06 | .06 | TP |
| Ln Irondose (mg/wk) | -.127 | .036 | **<.001** | / | / | / | / | / | / | VC |
| Ln Darbepoetin α (µg/wk) | .106 | .051 | **.04** | -.411 | .132 | .**002** | -.260 | .083 | **.002** | VC |
| Ln Epoetin β (IE/wk) | -.127 | 0.064 | .05 | / | / | / | / | / | / | VC |
| Ln CRP (mg/L) | .572 | .655 | .38 | / | / | / | / | / | / | CS |
| Ultrafiltration (L) | .270 | .110 | **.01** | -.261 | .145 | .07 | / | / | / | VC |
| Ln Phosphate (mmol/L) | -.080 | .037 | **.03** | / | / | / | / | / | / | VC |
| This table shows the model used for the analysis of the effects of the medication switch, iron deficiency at baseline, and anemia at baseline on all parameters, which are shown in the first column. The other columns show the fixed factors relevant for determining the effect of the switch on parameters, pre-switch being the reference category. Switch indicates the effect of switching from IS to FCM in all patients. Switch*A indicates the effect being anemic at baseline has on switching, in addition to the effect of the fixed factor switch, which is applicable in all patients. Switch*Idef indicate the same thing for patients iron deficient at baseline. There are four possible patient states for which the effect of the switch on parameters shown in the first column can be determined. For patients not iron deficient and not anemic, the effect in the second column is the full effect. For patients anemic but not iron deficient, add together the effects in the second and third column. For patients iron deficient but not anemic, add together the effects in the second and fourth column. For patients both iron deficient and anemic, add together the effects in all three columns. Covariance structures used in the model are described for each variable in the last column. A, anemic; Idef, iron deficient; SE, standard error; CS, compound symmetry; TP, Toeplitz; VC, variance components. | | | | | | | | | | |
